# Rewiring the future: drugs abused in adolescence may predispose to mental illness in adult life by altering dopamine axon growth

**DOI:** 10.1007/s00702-023-02722-6

**Published:** 2023-12-01

**Authors:** Radu Gabriel Avramescu, Giovanni Hernandez, Cecilia Flores

**Affiliations:** 1 Douglas Mental Health University Institute, McGill University, Montreal, QC, Canada; 2 Department of Psychiatry, McGill University, Montreal, QC, Canada; 3 Department of Neurology and Neurosurgery, Faculty of Medicine, McGill University, Montreal, QC, Canada; 4 Ludmer Centre for Neuroinformatics & Mental Health, McGill University, Montreal, QC, Canada

**Keywords:** Axon guidance, Axon targeting, Adolescence, Impulse control, Model organisms

## Abstract

Adolescence is a period of increased exploration and novelty-seeking, which includes new social behaviors, as well as drug experimentation, often spurred on by peer pressure. This is unfortunate, as the immature state of the adolescent brain makes it particularly susceptible to the negative developmental impact of drug use. During adolescence, dopamine terminals, which have migrated from the ventral tegmental area, pause in the nucleus accumbens, before segregating by either forming local connections or growing towards the prefrontal cortex (PFC). This developmentally late and lengthy process renders adolescent dopamine axon pathfinding vulnerable to disruption by substance use. Indeed, exposure to stimulant drugs in adolescent male mice, but not females, triggers dopamine axons to mistarget the nucleus accumbens and to grow ectopically to the PFC. Some evidence suggests that at this novel site, the functional organization of the ectopic dopamine axons mirrors that of the intended target. The structural rewiring dysregulates local synaptic connectivity, leading to poor impulse control ability, deficits of which are a core symptom of substance-use disorders. In the present commentary, we argue that different substances of abuse induce dopamine mistargeting events with the off-target trajectory prescribed by the type of drug, leading to psychiatric outcomes later in life.

## Introduction

Drug abuse rates are subject to fluctuations based on the region, the age of the users, and the drug in question; however, abuse of stimulants, opioids, and cannabis has generally increased in recent years (Drug Use Among Youth: Facts and Statistics 2023; Marijuana and hallucinogen use among young adults reached all time-high in 2021 2022; Drug Overdose Death Rates 2023; NIDA IC Fact Sheet 2024 2023). This situation has been accentuated in some areas due to changes in accessibility or regulation, such as the case of prescription opioids and the legalization of cannabis, respectively (Hughes et al. 2023; Sultan et al. 2023; Zuckermann et al. 2021).

The abuse of drugs during adolescence can have dire long-term mental health consequences. Adolescents, being at the perfect intersection of maturity to procure recreational drugs yet susceptible due to ongoing neurodevelopment, are particularly vulnerable to the negative effects of these substances, with worrisome repercussions. Adolescence is a period marked by increased exploration and novelty-seeking, encompassing new social experiences and often driven by peer pressure, which can lead to substance experimentation. Such experimentation significantly affects brain development, causing persistent neuroarchitectural changes. Of particular relevance is the mesocorticolimbic dopamine system, which plays a crucial role in reward processing and addiction and its dysregulation in response to drug use is tightly linked to increased impulsivity and compromised decision making, among others cognitive deficits (Volkow et al. 2019, 2002; Koob and Volkow 2016; Gulley and Juraska 2013; Dichter et al. 2012; Feltenstein and See 2008; Verdejo-García et al. 2008; Perry and Carroll 2008; Olmstead 2006; Jentsch and Taylor 1999). In this commentary, we propose that abuse of addictive drugs in adolescence can lead to ectopic innervation of dopamine axons to unintended areas, with implications for the development of psychiatric disorders later in life, particularly those that have at their core deficits in impulse control (Poulton and Hester 2019; Bakhshani 2014).

### The maturation of the mesocorticolimbic system during adolescence

The refinement in behavior and cognitive function occurring across adolescence is driven by the ongoing formation and maturation of neural connections, with many changes occurring in the mesocorticolimbic dopamine pathway (Arain et al. 2013; Herting et al. 2011; Peper et al. 2011; Walker et al. 2017; Meaney et al. 2002). In rodents, mesocorticolimbic dopamine terminals originating from the ventral tegmental area (VTA) and destined to innervate limbic or cortical regions undergo a late and lengthy pathfinding process. By the onset of adolescence, dopamine axons have already reached the nucleus accumbens and pause in this region before segregating into two distinct projections: those establishing local connections within the nucleus accumbens and those extending gradually towards the prefrontal cortex (PFC) (Reynolds et al. 2018; Hoops et al. 2018). This late-stage axonal growth sets it apart from most other monoamine systems, which achieve final long-distance wiring during embryogenesis or early postnatal life (Lidov et al. 1980; Levitt and Moore 1979).

Research in mice has shown that, at the molecular level, the guidance cue Netrin-1 and its receptor DCC (deleted in colorectal cancer) regulate the segregation of mesolimbic and mesocortical dopamine projections during adolescence. Changes in the expression of Netrin-1 or DCC during adolescent development lead to targeting errors and abnormal growth of dopamine terminals, and to the subsequent remodeling of local circuits in intended and off-target areas (Reynolds et al. 2018). Moreover, exposure to drugs of abuse in adolescence dysregulates the expression of the Netrin-1/DCC system in pre- and postsynaptic components of mesocorticolimbic dopamine neurons (Yetnikoff et al. 2011; Cuesta et al. 2018, 2020; Hernandez et al. 2022; Reynolds et al. 2023). This effect seems to be sex-specific, age and drug dependent for the few drugs that we have investigated.

### Exposure to amphetamine alters adolescent dopamine axon pathfinding

In adolescent mice, our results indicate that recreational, but not therapeutic-like doses of amphetamine downregulate both DCC receptors and Netrin-1 during early adolescence in males but not females (Cuesta et al. 2018, 2020; Reynolds et al. 2023). By late adolescence, this amphetamine regimen no longer regulates the Netrin-1/DCC system in males, while, in females, it now downregulates DCC levels and induces a compensatory increase in Netrin-1 (Reynolds et al. 2023). This causes mistargeting events in the nucleus accumbens and ectopic growth of mesolimbic projections to the PFC, which, by adulthood, establish aberrant synaptic connections and neurotransmission in this region (Reynolds et al. 2018). Amphetamine-induced structural rewiring of the adult PFC leads to impaired impulse control—a core symptom of several psychiatric disorders, including substance-use disorder (Poulton and Hester 2019; Kozak et al. 2019; Bakhshani 2014; Swann et al. 2008; Corruble et al. 2003; Moeller et al. 2001). Drug-induced alterations in dopamine axon pathfinding in adolescence may, therefore, contribute to the development of maladaptive behaviors, potentially setting the stage for increased susceptibility to psychiatric disorders. Ectopic dopamine axon growth is not observed in female mice exposed to amphetamine in adolescence. This sex-specific effect may result from differences in adolescent sensitive periods and/or from the engagement of compensatory processes (Reynolds et al. 2023), highlighting sexually dimorphic neurodevelopmental consequences of adolescent substance use.

### Dysregulation of dopamine system maturation in adolescence by drugs of abuse

Apart from amphetamine, our preclinical research also suggests a potential link between adolescent exposure to opioids and cannabis and axon mistargeting events. Adolescent mice exposed to oxycodone, a synthetic opioid which is currently in widespread use, show upregulation of *Dcc* mRNA expression in the dopamine system (G. Hernandez and C. Flores, unpublished observations). The direction of this change is opposite to the one induced by amphetamine and only occurs in males, suggesting that males are at risk of axon targeting errors. Other researchers have found indirect evidence of alteration of the maturation of the mesocorticolimbic dopamine system, with adolescent male mice exposed to morphine showing an increase in the mRNA expression of dopamine D1 receptors in the nucleus accumbens, 24 h after the last drug administration (Bates et al. 2018).

Adolescent mice exposed to tetrahydrocannabinol (THC), the main psychoactive component of cannabis, also exhibit alterations in the Netrin-1/DCC system in dopamine regions. *Dcc* mRNA is upregulated in the VTA in males, but downregulated in females, and only males show impulse control dysregulation in adulthood, suggesting compensatory effects in females, and male-specific risk of THC-induced dopamine mistargeting (Hernandez et al. 2022). THC exposure in adolescent mice alters the density and soma size of tyrosine-hydroxylase positive neurons in the adult VTA (Behan et al. 2012). In addition, in adolescent rats, THC exposure leads to increased spontaneous and burst firing of VTA neurons in adulthood, pointing to dysregulation of the maturation of the dopamine system (De Felice et al. 2021). We also note reviews that summarize additional findings of this dysregulation (Peters et al. 2021; Bloomfield et al. 2016).

Two additional drugs of abuse of significant importance are alcohol and nicotine, considering that they represent major and easily accessible drugs for teenagers. In mice, repeated ethanol administration during adolescence has been shown to lead to decreased ethanol-induced dopamine release in the nucleus accumbens and in the PFC in adulthood, in line with the idea that adolescent ethanol exposure may disrupt ongoing mesocorticolimbic dopamine development (Carrara-Nascimento et al. 2020). In addition, there is reduced expression of markers of dopamine axons in the PFC of adult rats that had intermittent ethanol exposure in adolescence, suggesting the possibility of altered innervation of dopamine axons due to axonal mistargeting (Trantham-Davidson et al. 2017).

In the case of nicotine, several researchers have shown, even if indirectly, alterations in dopamine wiring in the PFC of adult rodents exposed to nicotine during adolescence. For example, adolescent rats exposed to nicotine showed lower expression of dopamine D1 receptors in the PFC in adulthood, suggesting altered dopamine innervation (Jobson et al. 2019). Earlier experiments, using a similar paradigm of adolescent nicotine exposure in rats, showed changes in dopamine release in the PFC in adulthood (Counotte et al. 2009). Both these findings suggest that nicotine exposure during adolescence impacts the maturation of the dopamine system, possibly triggering errors in axonal pathfinding.

Administration of amphetamine during adolescence downregulates *Dcc* mRNA in male mice, but not females, while THC and oxycodone during adolescence upregulate *Dcc* mRNA in males, but not females. A possible explanation for these differential drug effects could be the way these drugs alter the firing rate of dopamine neurons. Studies done in adult rodents have shown that drugs of abuse affect the firing rate of dopamine neurons in both directions—some increase the frequency of action potentials, while others lower it. Amphetamine given in adulthood at a recreational-like dose decreases dopamine neuron firing (Valenti et al. 2021; Shi et al. 2000). Conversely, opioids and cannabis increase the firing of adult dopamine neurons (Jalabert et al. 2011; Melis et al. 2000; French et al. 1997; French 1997; Gysling and Wang 1983), with one study performed in adolescent male rats exposed to THC, corroborating the findings in adults (De Felice et al. 2021). These divergent effects may be associated with the discrepant effects on *Dcc* mRNA expression and are in line with reports showing that, in various model systems, DCC expression and its recruitment to growth cones, are regulated by action potential frequency, with higher frequency leading to higher expression and recruitment (Castillo-Paterna et al. 2015; Horn Katherine et al. 2013; Bouchard et al. 2008). Furthermore, drug-induced changes in DCC receptor expression in the VTA in adult rats require NMDA receptor activation (Yetnikoff et al. 2007). While activity-dependent regulation of DCC has not been shown in adolescent dopaminergic neurons, this mechanism has been observed in adult and embryonic neurons, making it more likely to be generalizable. We propose that any substance of abuse that affects the firing of dopamine neurons could alter DCC receptor expression and impact the segregation of pathway projections.

### Altered dopamine axon pathfinding in the clinical perspective

It is important to note that, in humans, the development of psychiatric pathology related to substance abuse depends on genetic predisposition, environmental factors, and the age at which drug use begins. For instance, a study found that Hispanic and White college students were more likely to report drug abuse than Asian or African American students, while males were overall more likely to report drug abuse than female students. This suggests an interplay between gender, ethnicity, and environment in the predisposition to developing a substance-use disorder (McCabe et al. 2007a). An additional illustration of genetic predisposition in response to drug abuse was noted in the case of cannabis, which is estimated to elevate the occurrence of psychotic symptoms by approximately 1.8-fold and may contribute to 8–14% of schizophrenia cases (Moore et al. 2007; Henquet et al. 2005; Fergusson et al. 2003). Beyond genetic and environmental considerations, the age of onset of drug use also exerts significant influence on the development of substance use disorders. A study involving individuals aged 13–21, encompassing various classes of prescription drugs with abuse potential, revealed that delaying the initiation of drug use by 1 year decreased the odds of developing lifetime prescription drug abuse by 5% (McCabe et al. 2007b). These studies and others (Hines et al. 2015; Milaniak et al. 2015; Enoch 2012) illustrate the interaction between genetics and environment in the predisposition to initiate drug abuse and the subsequent development of psychiatric pathology.

Drug-induced alterations in dopamine axon pathfinding during adolescence may be an essential component of harmful mental health consequences, and in this regard, there may be noticeable differential responses to the same drug experience between males and females. Clinically, a large population study from Denmark found that for psychiatric disorders due to substance abuse, the lifetime risks were 7.79% for men and 4.49% for women, which are also associated with a significantly higher incidence in men (Pedersen et al. 2014). The same group looked at psychiatric disorders, including substance-use disorders, in adolescents, and while there were many differences overall, the cumulative incidence of substance use disorder in girls was 1.53% versus 1.63% for boys, which is a negligible distinction (Dalsgaard et al. 2020). These two studies suggest that while girls and boys start with a similar predisposition to substance-use disorders, by adulthood, men are almost twice as likely to develop disorders arising from substance abuse. The specific factors accounting for these sex differences are unknown. However, these reports are in general agreement with our findings in mice, showing predisposition for male adolescents to exhibit dopamine axon mistargeting in response to exposure to drugs of abuse.

### Potential interaction of drugs of abuse in adolescence and circulating gonadal hormones

We speculate that the main candidates involved in sexual dimorphic effects of drugs of abuse on adolescent dopamine axon pathfinding may be circulating sex hormones, the differential expression pattern of sex hormone receptors in the brain, and sex-specific timing in adolescent sensitive periods. Sex hormones on their own have long been recognized to reorganize neuronal circuits during adolescence (Herting et al. 2011; Peper et al. 2011; Vigil et al. 2016; Arain et al. 2013) and testosterone exposure has been shown to increase the diameter and anterograde vesicular transport of axons in rats (Pesaresi et al. 2015). Studies in invertebrate organisms show that the control of DCC and Netrin-1 expression during the juvenile period is sexually dimorphic, leading to the development of sex-specific synaptic networks (Weinberg et al. 2018).

Findings from Kritzer and Creutz (2008) reported that adult male rats have about half the number of VTA-PFC projecting dopamine neurons than females, suggesting sex differences in the extent of the segregation of mesolimbic and mesocortical dopamine pathways. In addition, McArthur et al. (2007) observed that adult female rats have approximately 30% more neurons in the VTA and a 50% larger overall VTA volume. Immunostaining for androgen and estrogen receptors in the VTA of adult rats showed that males have significantly more dopaminergic neurons expressing either androgen or estrogen receptors, with females having relatively few neurons expressing these receptors. Work done on adult female mice during estrus, a time of high circulating estrogen, showed a large increase in the activity of dopamine neurons in the VTA, when compared to males or females in diestrus (Calipari et al. 2017). This suggests that although the female VTA expresses relatively lower levels of estrogen receptor, high circulating estrogen still has a prominent effect on the activity of neurons in this area. These studies, as well as our own work, raise the tantalizing possibility of the interaction of drugs of abuse in adolescence with sex steroid specific effects, which may help explain differential predisposition to pathology of adolescent males versus females in response to drugs of abuse.

## Conclusion

Adolescence is a vulnerable period characterized by heightened drug experimentation and additional susceptibility to persistent neurodevelopmental effects of drugs of abuse. The intricate process of dopamine axon pathfinding occurring at this age renders the developing brain particularly sensitive to the effects of substance use, leading to changes in brain circuitry and behavior in adulthood. By elucidating the molecular, cellular, and hormonal underpinnings of these alterations, we can better predict psychiatric outcomes. Ultimately, this knowledge could pave the way for targeted interventions aimed at minimizing the long-term impact of adolescent drug use on mental health.

## Data Availability

Not applicable.
